# A Focus on the Optical Properties of the Regenerated Newt Lens

**DOI:** 10.1371/journal.pone.0070845

**Published:** 2013-08-22

**Authors:** Sarah Wassmer, Margaret Beddaoui, Payman Rajai, Réjean Munger, Catherine Tsilfidis

**Affiliations:** 1 Ottawa Hospital Research Institute, Vision Research Program, Ottawa, Ontario, Canada; 2 Department of Cellular and Molecular Medicine, University of Ottawa, Ottawa, Ontario, Canada; 3 Department of Physics, University of Ottawa, Ottawa, Ontario, Canada; 4 Department of Ophthalmology, University of Ottawa, Ottawa, Ontario, Canada; University of Dayton, United States of America

## Abstract

Lens regeneration studies in the adult newt suggest that molecular aspects of lens regeneration are complete within 5 weeks of lentectomy. However, very little is known about the optical properties of the regenerated lens. In an aquatic environment, the lens accounts for almost all of the refractive power of the eye, and thus, a fully functional lens is critical. We compared the optical properties of 9- and 26-week regenerated lenses in the red spotted newt, *Notophthalmus viridescens*, with the original lenses removed from the same eyes. At 9 weeks, the regenerated lenses are smaller than the original lenses and are histologically immature, with a lower density of lens proteins. The 9 week lenses have greater light transmission, but significantly reduced focal length and refractive index than the original lenses. This suggests that following 9 weeks of regeneration, the lenses have not recovered the functionality of the original lens. By 26 weeks, the transmission of light in the more mature lens is reduced, but the optical parameters of the lens have recovered enough to allow functional vision.

## Introduction

Lens regeneration in the newt, *Notophthalmus viridescens*, results from the dedifferentiation and proliferation of cells of the pigmented iris epithelium. This transdifferentiation of iris to lens is found in several urodele amphibian species, and has been known for over a hundred years, since it was first documented by Colluci [1] and later Wolff [2]. The cellular events of lens regeneration have been extensively studied, and excellent comprehensive reviews are available [3]–[9]. These studies, and others, show that the removal of the lens leads to rapid structural changes in the iris epithelial cells (IECs). IECs of the dorsal iris undergo depigmentation and re-enter the cell cycle. The inner and outer laminae of the iris epithelium separate, and depigmented cells continue to proliferate at the mid-dorsal margin of the iris epithelium, leading to the formation of the lens vesicle. As depigmented cells are continuously added to the outside anterior part of the lens vesicle, there is elongation and differentiation of lens fibres in the inner posterior layer. The expression of lens proteins (crystallins), the differentiation of primary and secondary lens fibres and the formation of the lens capsule ensue. In the final stages, the lens detaches from the iris. Eguchi et al [10] showed that the lens regeneration process proceeds perfectly irrespective of the age of the animal, and that one animal can be lentectomized multiple times without compromising the timing or the flawlessness of the regeneration process.

The lens regeneration process is divided into 11 or 13 stages, depending on the species of newt involved [3]–[5], [11]. There is some variability in the timing of these stages, which can be attributed to the temperature of the environment, the species or subspecies of newt involved and the surgical procedure of lens removal. It is generally reported that most stages are completed by 30–35 days after lentectomy [3], [5]. Inoue et al [12] showed that regeneration in larval and adult animals progresses at the same speed, and most of the stages are complete within one month after lentectomy, based on crystallin gene re-expression. Stone and Steinitz [13] examined hundreds of lenses for 60 days after lentectomy, and found they had completed stage 12 and redifferentiated by this timepoint. However, the final stage (stage 13), involving the continued growth of the lens can last for a long time. Furthermore, the timing of complete regeneration, including growth to the original size, varies significantly depending on the study. Stone and Chace [14] suggested that the regenerated lens doesn't attain the size of the original lens, even after a one-year follow-up in *Notophthalmus viridescens*. Eguchi et al [10] reported that the lens in the Japanese newt, *Cynops pyrrhogaster,* attains the original size within 5 months of lentectomy.

Light is focused on the back of the eye, the retina, by two main structures – the transparent external cornea and the transparent lens. The cornea and lens are both important focussing elements of the eye in air. However, in water, the cornea contributes very little to refraction. In aquatic species such as *Notophthalmus viridescens*, the lenses are spherical in shape. The lens does not undergo the flattening which is associated with metamorphosis and movement to land found in some other amphibians [15], [16]. Consequently, the lens has a high refractive index and is very important for focusing light on the retina. As a result, the optical properties of the lens, including clarity, transmission and focal length are critical. Previous studies on lens regeneration have focused almost entirely on the cell biology and histology of the regenerated lens. To date, the optical properties of the regenerated lens, and the ability of the lens to support functional vision have not been assessed. In the current study, we compare transmission and focal length in original and regenerated lenses at 9 weeks and 26 weeks after lentectomy to determine if the newt regains full functional vision after lens regeneration.

## Results

*Notophthalmus viridescens* adult newts were lentectomised and the extracted lenses were assessed for size, transmission, focal length and refractive index. The animals were allowed to regenerate for 9 or 26 weeks, and then re-lentectomised. The properties of the regenerated lenses were determined and compared to the original intact lenses.

The 9- and 26-week timepoints were chosen based on previous lens regeneration studies. Most studies suggest that all stages (except for the final growth stage) are complete by 30–35 days after lentectomy. We chose 9 weeks post-lectectomy because this timepoint should be well past the completion of the molecular pathways of the regeneration process. We also chose 26 weeks because a recent study on lens regeneration in the *Cynops pyrrhogaster* newt suggests that the lens is fully grown by 5 months after lentectomy.

The lens continues to grow as the animal ages, and so the size and the focal length of the lens will depend on the age of the animal. Since the animals in this study were collected from the wild and their age is not known, regenerated lenses were compared to the control lenses removed from the same animals in order to ensure that the relative age of the groups was similar. Thus, any differences in the measurements of size and focal length could not be accounted for by differences in age between the two groups.

### 9-week regenerates

**Figure 1:**
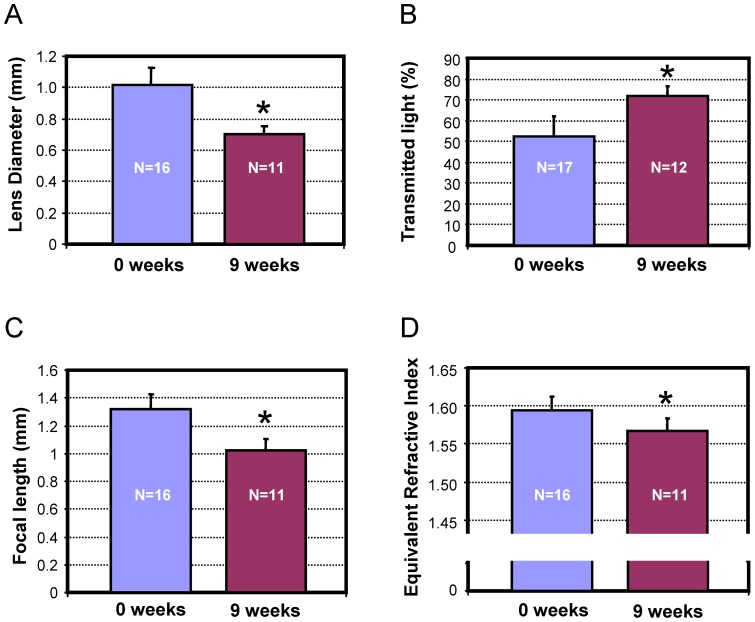
The optical properties of the original and 9-week regenerated lenses. A) Lens diameter is significantly reduced in 9-week regenerates. B) Transmitted light of regenerated lens is higher than original lens. This is often typical of younger lenses. C) Focal length of regenerated lens is lower than original lens. D) Lens equivalent refractive index is significantly lower in regenerated the lens.

In healthy adult newts, the normal lens is approximately 1 mm in diameter. The median for our group was 0.998 mm. After 9 weeks of regeneration, most regenerated lenses were <0.80 mm in diameter (median = 0.705, Figure 1A), a statistically significant difference (P<0.001). Individual values are presented in the supplemental material (Table S1).

Optical functionality of the original and the regenerated lenses at 9 weeks was also found to have significantly different parameters. The lenses extracted from the healthy adult newts had a median transmitted light of 53% while the regenerated lenses transmitted significantly more light (P<0.001) with a median value of 73% (Figure 1B, Table S1). This is consistent with the expected higher transparency of younger lens material [17]–[19].

The mean calculated focal length in the eye of the crystalline lens of our group of healthy adult newts was 1.32 mm (Figure 1C, Table S1). The mean calculated focal length in the eye of the regenerated lenses after 9 weeks was significantly (P<0.001) smaller (by 0.297 mm or 22.5%) than the original lenses extracted from the same eye (Figure 1C). This is consistent with the smaller radius of curvature of the lens assuming a fully formed gradient of refractive index.

The median calculated equivalent homogeneous index of refraction of the lenses extracted from the healthy adult newts was 1.59 while that of a 9-week regenerated lens was 1.56, a statistically significant (P<0.001) difference (Figure 1D, Table S1). This suggests that the gradient of refractive index in the regenerated lens is not fully developed.

**Figure 2:**
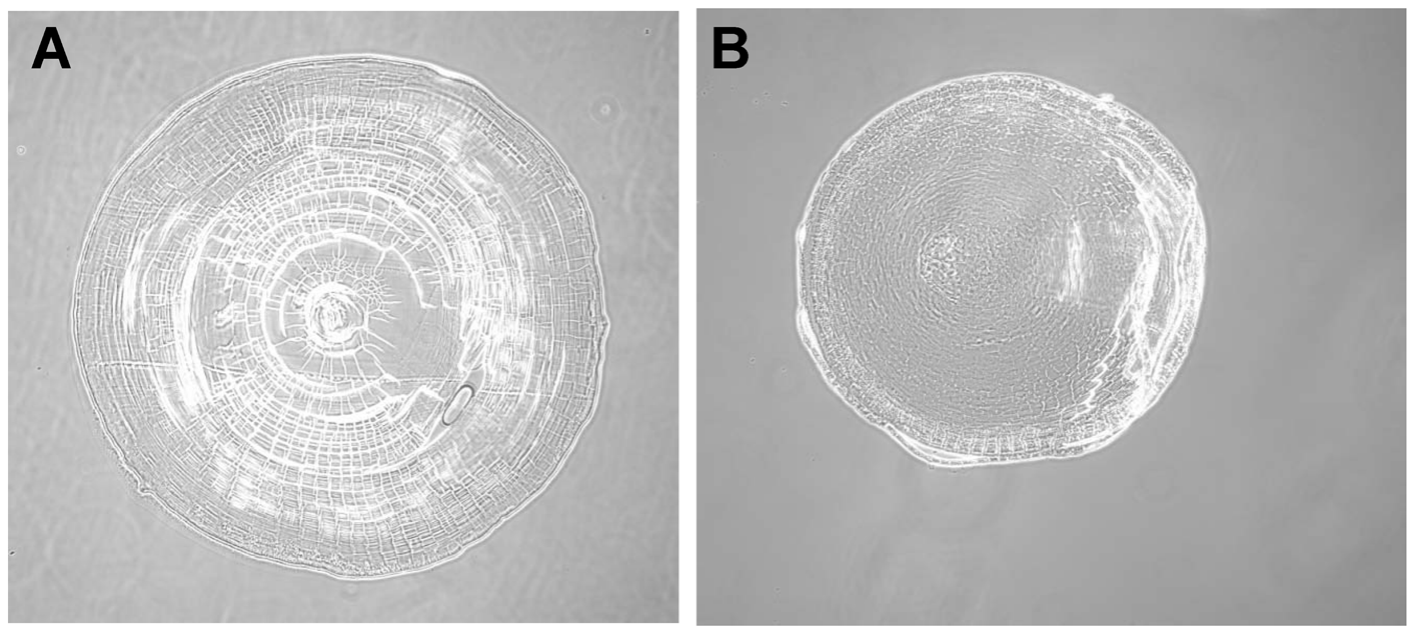
Histology of the original and 9-week regenerated lenses. The regenerated lens (B) is smaller and less compact than the original lens (A) and does not have a distinct nucleus and well developed cortex.

**Figure 3:**
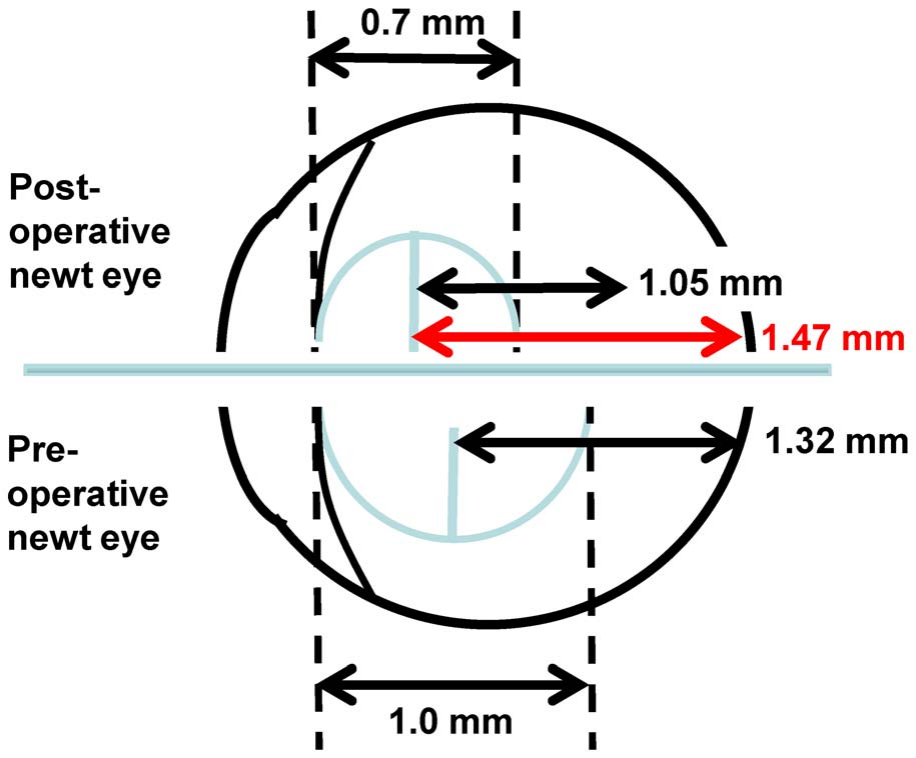
Schematic of newt eye (drawn to proportion from Litzinger and Del Rio Tsonis [**23**] **).** The original (pre-operative) lens is shown on the bottom. Based on ocular anatomy, a predicted focal length of 1.32 would ensure a clear image is focused on the retina. The 9-week regenerated lens, with its smaller diameter and shorter focal length of 1.05 mm would focus the image in front of the retina, resulting in a blurred image. The focal distance would have to be 1.47 in the smaller diameter lens in order to have proper functionality.

Histologically, the 9-week regenerated lenses were less dense than their original counterparts, and did not have well developed nuclei and cortical structures (Figure 2). This, combined with the reduced size, reduced focal length, and reduced index of refraction suggest that after 9 weeks, the regenerated lens had not yet recovered the functionality of the original lens. The 9 week lens would focus the image in front of the retina, resulting in a blurred image (Figure 3).

### 26-week regenerates

As the 9- and 26-week protocols differed in their “control”, the first priority was to validate the equivalence of the control groups for the 9-week and the 26-week control eye lenses (Tables S1 and S2). On average the 26-week control lenses were slightly larger (0.09 mm) than the 9-week control lenses but the difference was not statistically significant (P = 0.104). Similarly, the focal length in the eye (P = 0.447) and equivalent refractive index (P = 0.160) were not statistically different between the two “control” groups.

**Figure 4:**
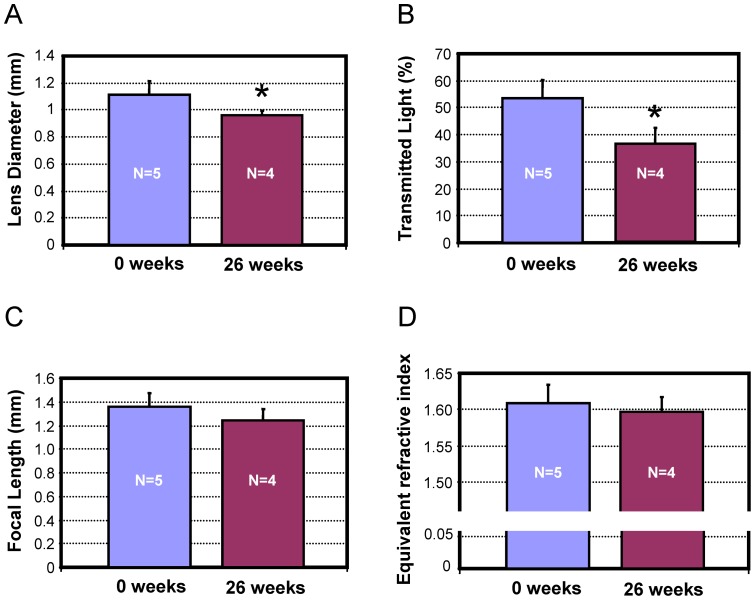
The optical properties of the original and 26-week regenerated lenses. A) Lens diameter is still significantly smaller in the regenerated lens, but the difference between the regenerated and original lenses is much smaller than at 9 weeks. Focal length (B) and refractive index (C) are similar in control and regenerated lenses. D) The transmitted light for the regenerated lens is significantly lower than the original lens, but should not significantly impact functional vision in the newt.

**Figure 5:**
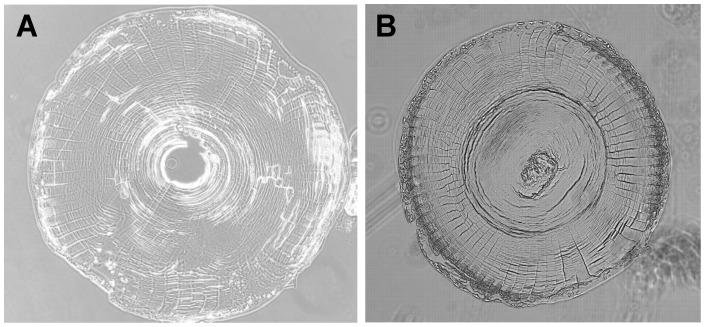
Histology of the original and 26-week regenerated lenses. The regenerated lens (B) is still smaller than the original lens (A), but is now much more compact and has mature differentiated structures similar to the intact lens.

On average the 26-week regenerated lenses were significantly smaller (by 0.146 mm, Figure 4A, P = 0.026) than their original controls. Individual values are presented in the supplemental material (Table S2). Compared to the 9-week lenses (difference in median of 0.293) the difference between 26-week regenerated lenses and controls had decreased significantly (P<0.001). At 26 weeks the regenerated lenses transmitted significantly (P = 0.006) less light (by 17.3%) than the control lenses, the opposite of the results found for the lenses after 9 weeks of regeneration (Figure 4B). The differences in size and transmission did not translate into a difference in lens functionality. The calculated focal lengths in the eye (figure 4C, P = 0.138) and equivalent indices of refractions (Figure 4D, P = 0.448) were statistically the same for the 26-week regenerated and control lenses. Histologically, the lenses had developed mature features, and were virtually indistinguishable from the original lenses (Figure 5).

These results suggest that after 26 weeks of regeneration, the lens is fully functional, even if it is slightly less transparent and smaller than the original lens.

## Discussion

The ability of the red-spotted newt to regenerate limbs and organs is well established. To be useful the regenerated tissue must not only replace the absent tissue, it must also be able to perform its original function. Previous work has established that the newt can regenerate its crystalline lens. The structural aspects of this regeneration process have been well characterized.

In an aquatic environment, the lens accounts for almost all of the refractive power of the eye. For this reason, the regeneration of both the structural components *and* the functional capacity of the lens are critical to ensure visual recovery. To be fully functional, the regenerated lens must reproduce the gradient of refractive index in order to provide the optical quality required to match the original visual capabilities of the newt.

In this paper we investigated the optical function and the morphology of normal and regenerated lenses to establish the functional capabilities of the regenerated crystalline lens. The lenses of healthy adult newts transmitted approximately 55% of the incident light while in mineral oil. This value is likely lower than the actual transmitted light in the eye because of the higher index of refraction difference at the lens/oil interface (1.36/1.48) than at the lens /water interface (1.36/1.33) found in the eye. We did not correct for this index difference because we do not know the actual lens surface index and because of the influence of the lens's spherical surface on the amount of light specularly reflected. As demonstrated in Figures 1C and 4C, the calculated focal length of the control lens is consistent with the predicted values based on known ocular anatomy (see Figure 3). Previous work [20] with spherically symmetric lenses has used the focal length to lens radius (f/R) to compare the lens power across species. Our healthy adult newt lenses had a mean f/R of 2.56 which is consistent with other species. This would suggest a similar gradient of refractive index (GRIN) for the newt as for other species such as the African cichlids [20]. A recent paper by Hoshino et al [21] measuring the GRIN in several species, including the newt, shows profiles consistent with this conclusion. Since one of the properties of the gradient is to produce an effective homogeneous refractive index greater than the core index (for example the core index of the human lens is 1.40 while the equivalent refractive index is 1.42), our result of a mean homogeneous refractive index of 1.59 is also consistent with known properties of the GRIN for small spherical lenses.

At 9 weeks we find that the lenses are significantly smaller (by 20%) than the original lenses. This is consistent with earlier work by Stone and Chace [14] showing that in *Notophthalmus viridescens,* the regenerated lens takes a long time to approach its original size. The 9-week old lenses also have a significantly shorter focal length than the original lenses. As shown in figure 3, this indicates that the lens cannot create a well-focused image on the retina and the eye is not fully functional. At 9 weeks, the lens f/R ratio is significantly larger than that of the original lens (Table S3). This means that it has less power than would be expected if it had a fully developed GRIN, suggesting that the lens is not fully matured morphologically. This is confirmed by the cross-sectional appearance of the lens which shows a less compact formation with no distinct nucleus or cortical structures. Finally, at 9 weeks the regenerated lens transmits significantly more light than the original lens. The smaller size of the lens means a shorter optical path that will reduce light losses in the lens and thus slightly increase transmitted light. The large increase in transmitted light suggests that in addition to the size effect, the newly formed lens material is more transparent than the original lens material. This would be consistent with the lower protein density in the lens that is seen in the cross-sections and that would accompany the lower refractive index of the regenerated lens.

At 26 weeks, the histology of the regenerated lens resembles the original lens. Even at this stage, however, we found that the diameter of the lens is still smaller than the original lens, in agreement with Stone and Chase [14]. All other results suggest that the regenerated crystalline lens is functionally equivalent to an original lens except for its lower light transmission. We cannot at this time explain why the lens has a lower transmission. The impact of this lower transmission should not significantly impact the vision quality of the newt as it is not scattered light which would show up as a veiling light on the retina and would result in reduced image contrast. If we assume that newts, as do most animals, can adjust their retinal sensitivity based on luminance, then they should easily compensate for the loss in image brightness without significant loss in visual performance.

## Conclusions

Overall, we show that previous studies which suggest that most stages of lens regeneration are complete by 5 weeks following lens loss may be accurate for molecular aspects of regeneration, but they do not take into account functional measures. We show that even at 9 weeks, the regenerated lens forms a blurred image on the retina, and it takes a full 26 weeks or more for the newt to regain full functional vision.

## Materials and Methods

### Lentectomy Procedure

Adult newts were purchased from Charles D. Sullivan Co, Inc. (USA). They were kept at 22°C in large aerated tanks with running dechlorinated water on a 12 h light/12 h dark cycle. Newts were fed live blackworms twice weekly. All experimental procedures were approved by the University of Ottawa Animal Care and Veterinary Service (Permit number: EI-20). Prior to lentectomy, newts were anaesthetised for 15 minutes in 0.05% MS-222 (tricaine methanesulfonate; Sigma), buffered with sodium hydrogen carbonate (BDH Inc.). The newts were then placed on a Petri dish lined with an MS-222-soaked cheese cloth. Pictures were taken of the skin spot pattern of each newt using a Zeiss Dissecting Microscope for identification purposes. For the lentectomy, a small vertical incision was made in the ventral cornea with a 30 g needle. Microsurgery scissors were used to extend the corneal incision dorsally. Forceps were used to place pressure on either side of the incision to extract the lens. The lens was immediately placed in a 35 mm glass-bottom dish (MatTek Corp.) containing 2 mL of mineral oil (ACP Chemicals Inc.). The lens was then transported to the Transmission and Scatter Measuring System (TSMS).

### Transmission and Scatter Measuring System (TSMS)

A custom device was built in one of the authors' laboratories (RM) to measure the interaction of light with biological samples. The apparatus is designed to measure simultaneously the forward scattered, the back scattered and the transmitted light following interaction with a sample. Transmitted light measurements capture all light passing through the sample and exiting within 5° of the incident beam direction, whereas the scattered light captures light within the 2.5 to 25° region around the incident (back scatter) and transmitted beam (forward scatter) direction. As scatter signals were very small and did not change significantly across all tests we will limit our discussion to the transmitted light measurement. The transmitted light measurements are presented as the percentage of the light incident on the sample that exits within the capture area of the detector (±2.5°). It thus contains signal not absorbed by the sample but also some low angle scatter signal and that is why we refer to it as the transmitted light.

For all measurements the sample was positioned in a plane conjugate with an aperture controlling the diameter of the beam (but not conjugate with the light source) to ensure a uniform illumination of the sample and a well-defined area of illumination controllable by varying the aperture size. For the experiments discussed in this paper the aperture was selected to produce an illuminated area 0.5 mm in diameter at the sample. The device measures the incident (Vi) and transmitted light (Vt) intensities simultaneously to compensate for any inherent light source fluctuations. The device provides the intensity data as voltages that must be converted into transmitted signals using the following steps:

- Correct all voltages for dark current (for incident, Ni, and transmitted, Nt, signals). The dark current is the relatively small current that flows through any light detecting device (in our case a photo-diode), even when no light is incident on it. Dark current needs to be measured for each device as it is unique to each due to small variations in manufacturing.
- Obtain the ratio of transmitted to incident signal R  =  (Vt-Nt)/(Vi-Ni)
- Use a calibration (discussed below) of Transmitted (%) vs R to extract transmitted value

The transmitted signal is calibrated by obtaining the R-values for a series of calibrated interference filters (0.1 to 3.0 Optical Density). Calibration is performed every time a new beam diameter is used, a light bulb is changed or the device has not been operated for more than a week. In this case a new calibration was performed before each set of experiments at either the pre- or post-regeneration time points.

The protocol for transmitted light measurement for each time point was thus:

- Calibrate the transmitted signal with interference filters
- Measure signals without any light (electronic noise)
- Measure signals without sample (100% transmitted signal)
- Measure signals for test conditions

Samples were placed in a Petri dish filled with 2 ml of mineral oil so that introducing the lens did not affect significantly the volume of oil the test beam had to traverse with or without a lens in its path. The mineral oil provided an anoxic environment that preserved the clarity of the lens during the testing procedure. The bottom of the Petri dish is a thin coverslip glass to ensure maximum transmission and minimum contribution from the dish. Three measurements were performed for each sample, with the sample removed from the device and realigned with the test beam for each of the measurements. Alignment with the beam positioned the beam over the center of the sample as per visual inspection with a 4x magnifier. As the oil/dish combination is not completely transparent (loss of 14.5% due to reflection from interfaces and absorption by the oil), all transmitted light measurements provided in the rest of this paper have been corrected for these losses. All Petri dish/oil combinations were measured without samples to ensure proper correction for their individual contribution to the final lens measurements. TSMS measurements were taken at exactly 3 minutes after lentectomy to control for possible changes in transparency and transmission with time.

### Focal Length Measurements

**Figure 6:**
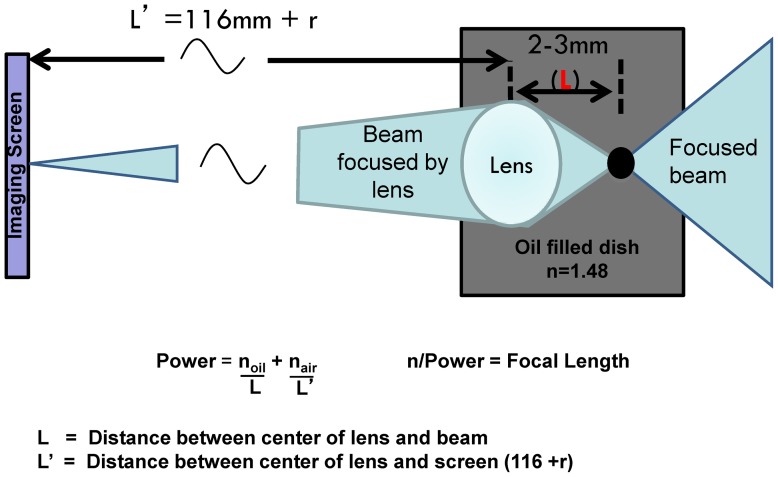
Focal length measurements setup. A collimated red HeNe laser beam 10 mm in diameter is focused by a long working distance objective lens (Mitutoyo, 40x) positioned on a graduated translational stage (±0.05 mm) such that the focus point can be moved relative to the lens until it produces a focus point on a screen 116 mm from the bottom of the dish. The power and focal length of the lens can be calculated using the formulas shown.

Focal length measurements were performed within 30 minutes of the transmitted light measurements with the lens remaining in the original oil-filled Petri dish. The measurement was performed as per Figure 6 where a collimated red HeNe laser beam 10 mm in diameter is focused by a long working distance objective lens (Mitutoyo, 40x) positioned on a graduated translational stage (±0.05 mm) such that the focus point can be moved relative to the lens until it produces a focus point on a screen 116 mm from the bottom of the dish.

The newt lenses are spherical so their principal planes are at the center of the lens [21]. All focal length values are thus reported as distances from the lens center to the focal point. Since the original lens power is measured in oil, it must be converted to power in the newt eye. The conversion is done using a simple thin lens model using refractive indices n_o_ = 1.48 for the mineral oil and n = 1.33 for the aqueous of the newt eye.

The refractive index of the newt crystalline lens has not been measured but like all spherical lenses measured in other species [22] it is expected to have a gradient of refractive index where the index increases gradually to its maximum at the lens center. This construct allows the lens more focusing power than could be achieved with a homogeneous refractive index equal to the index at the lens center. To test this assumption, the concept of an equivalent homogeneous refractive index (n_e_) is often used. This index is the homogeneous refractive index that would be required to give the lens the power measured. The n_e_ is calculated using the Lensmaker's equation with the surface curvatures equal to the lens radius, r, the lens focal length in oil, f, and the index of the oil, n_o_ = 1.48:


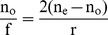
 **or** 
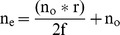


### Lens Size Measurements

Lenses were imaged using the Zeiss Dissecting Microscope. *AxioVision Rel. 4.8* software was used to place a box around the lens, and the length and width of the box were determined using a micron ruler. In lenses where the length and width were not exactly equal, an average of the two was taken.

### Histological Preparation

Lenses were fixed with 4% paraformaldehyde overnight at 4°C, and washed three times with 1x PBS. They were incubated in 30% sucrose for 48 hours, and then equilibrated in a 1∶1 solution of Optimum Cutting Temperature (OCT; Tissue-Tek*®*) and 30% sucrose in 1xPBS overnight. Lenses were frozen in the OCT/30% sucrose mixture over liquid nitrogen for a minimum of five minutes and stored at −80°C. They were sectioned at a maximum thickness of 10 μm using a Shandon Cryotome FSE (Thermo electron Corp), and transferred to Superfrost Plus slides (Fisherbrand, Fisher Scientific). Sections were washed with one drop of distilled water, left to dry overnight and mounted with a cover slip (Fisherbrand) using glycerol. The total number of sections per lens was counted to accurately identify the center of the lens. All images were taken at the center of the lens.

### Statistical Analysis

All group comparisons were made using a 2 tailed t-test if the data was normally distributed or a Mann-Whitney Rank Sum test if not normally distributed, using 95% confidence intervals.

## Supporting Information

Table S1 **Lens characteristics for the 9-week regenerates.**
(DOC)

Table S2 **Lens characteristics for the 26-week regenerates.**
(DOC)

Table S3 **Ratio of focal length to radius for control and regenerated lenses.**
(DOC)
