# Supplementary material for: A Focus on the Optical Properties of the Regenerated Newt Lens
Source: PLoS One. 2013 Aug 22;8(8):e70845. doi: 10.1371/journal.pone.0070845 (PMC3750027; doi:10.1371/journal.pone.0070845)
Supplement: Table S1 — Lens characteristics for the 9-week regenerates. (DOC) [file pone.0070845.s001.doc]

Supplementary Table S1 – Lens characteristics for the 9-week regenerates

| **Lens Number** | **Diameter (mm)** | | **Refractive Index** | | **Focal Length (mm)** | | **Transmission (%)** | |
| --- | --- | --- | --- | --- | --- | --- | --- | --- |
|  | Time 0 | 9 weeks | Time 0 | 9 weeks | Time 0 | 9 weeks | Time 0 | 9 weeks |
|  |  |  |  |  |  |  |  |  |
| 1 | 1.000 |  | 1.609 |  | 1.223 |  | 61.8 |  |
| 2 | 0.950 | 0.745 | 1.589 | 1.561 | 1.255 | 1.108 | 55.2 | 67.7 |
| 3 | 1.070 |  | 1.594 |  | 1.387 |  | 52.7 |  |
| 4 | 1.030 | 0.750 | 1.576 | 1.609 | 1.431 | 0.918 | 41.1 | 76.8 |
| 5 | 0.875 | 0.735 | 1.585 | 1.576 | 1.176 | 1.023 | 52.0 | 76.5 |
| 6 | 0.995 |  | 1.581 |  | 1.356 |  | 47.5 |  |
| 7 | 1.065 |  | 1.582 |  | 1.447 |  | 59.7 |  |
| 8 | 1.300 |  | 1.610 |  | 1.583 |  | 37.6 |  |
| 9 |  | 0.680 |  | 1.552 |  | 1.051 | 69.8 | 71.3 |
| 10 | 0.960 | 0.735 | 1.581 | 1.560 | 1.310 | 1.098 | 40.6 | 61.5 |
| 11 | 0.970 | 0.735 | 1.584 | 1.570 | 1.304 | 1.050 | 65.6 | 73.1 |
| 12 | 1.060 | 0.705 | 1.615 | 1.576 | 1.267 | 0.982 | 56.3 | 76.9 |
| 13 | 0.985 | 0.685 | 1.586 | 1.562 | 1.317 | 1.014 | 49.3 | 67.9 |
| 14 | 1.000 | 0.695 | 1.590 | 1.560 | 1.313 | 1.035 | 48.1 | 73.6 |
| 15 | 1.195 | 0.705 | 1.646 | 1.547 | 1.289 | 1.116 | 34.8 | 70.6 |
| 16 | 0.925 | 0.565 | 1.586 | 1.565 | 1.234 | 0.824 | 61.1 | 75.1 |
| 17 | 0.945 |  | 1.603 |  | 1.181 |  | 54.5 | 72.6 |
|  |  |  |  |  |  |  |  |  |
| Mean | 1.020 | 0.703 | 1.595 | 1.567 | 1.317 | 1.020 | 52.2 | 72.0 |
| St Dev | 0.105 | 0.052 | 0.018 | 0.016 | 0.106 | 0.087 | 9.9 | 4.6 |
| Median | 0.998 | 0.705 | 1.588 | 1.562 | 1.307 | 1.035 | 52.7 | 72.8 |
