# Supplementary material for: A Focus on the Optical Properties of the Regenerated Newt Lens
Source: PLoS One. 2013 Aug 22;8(8):e70845. doi: 10.1371/journal.pone.0070845 (PMC3750027; doi:10.1371/journal.pone.0070845)
Supplement: Table S2 — Lens characteristics for the 26-week regenerates. (DOC) [file pone.0070845.s002.doc]

Supplementary Table S2 – Lens characteristics for the 26-week regenerates

| **Lens Number** | **Diameter (mm)** | | **Refractive Index** | | **Focal Length (mm)** | | **Transmission (%)** | |
| --- | --- | --- | --- | --- | --- | --- | --- | --- |
|  | Time 0 | 26 weeks | Time 0 | 26 weeks | Time 0 | 26 weeks | Time 0 | 26 weeks |
|  |  |  |  |  |  |  |  |  |
| 10 | 0.945 | 0.950 | 1.585 | 1.614 | 1.267 | 1.141 | 57.0 | 30.3 |
| 11 | 1.125 | 1.005 | 1.581 | 1.611 | 1.537 | 1.222 | 50.1 | 44.2 |
| 12 | 1.205 | 0.960 | 1.622 | 1.569 | 1.407 | 1.376 | 42.7 | 34.5 |
| 13 | 1.115 |  | 1.624 |  | 1.295 |  | 58.0 |  |
| 14 | 1.165 | 0.945 | 1.637 | 1.594 | 1.294 | 1.222 | 59.4 | 35.7 |
|  |  |  |  |  |  |  |  |  |
| Mean | 1.111 | 0.965 | 1.610 | 1.597 | 1.360 | 1.240 | 53.5 | 36.2 |
| St Dev | 0.099 | 0.027 | 0.025 | 0.021 | 0.112 | 0.098 | 7.0 | 5.8 |
| Median | 1.125 | 0.955 | 1.622 | 1.603 | 1.295 | 1.222 | 57.0 | 35.1 |
