# Supplementary material for: A Focus on the Optical Properties of the Regenerated Newt Lens
Source: PLoS One. 2013 Aug 22;8(8):e70845. doi: 10.1371/journal.pone.0070845 (PMC3750027; doi:10.1371/journal.pone.0070845)
Supplement: Table S3 — Ratio of focal length to radius for control and regenerated lenses. (DOC) [file pone.0070845.s003.doc]

Supplementary Table S3 – Ratio of focal length to radius for control and regenerated lenses

|  | **Diameter (mm)** | **Focal Length (mm)** | **f/R** |
| --- | --- | --- | --- |
|  |  |  |  |
| Time 0 control (n=21) | 1.042 | 1.327 | 2.55 |
|  |  |  |  |
| 9 week regenerate (n=11) | 0.703 | 1.020 | 2.90 |
|  |  |  |  |
| 26 week regenerates (n=4) | 0.965 | 1.240 | 2.57 |
